# Leveraging Artificial Intelligence in Breast Cancer Screening and Diagnosis

**DOI:** 10.7759/cureus.79177

**Published:** 2025-02-17

**Authors:** Abdul Haseeb Hasan, Umar Abdul Rehman Khalid, Muhammad Ali Abid

**Affiliations:** 1 Medicine, Mayo Hospital, Lahore, PAK; 2 Medicine, King Edward Medical University, Lahore, PAK

**Keywords:** artificial intelligence, breast cancer, convolutional neural networks (cnn), deep learning (dl), machine learning (ml), mammography, screening, ultrasound

## Abstract

Breast cancer remains the most prevalent malignancy worldwide, posing a significant public health burden due to its high incidence and mortality rates. Early detection through mammography has been instrumental in reducing breast cancer-related deaths; however, traditional screening methods are constrained by human limitations, including variability in interpretation and resource-intensive workflows. Artificial intelligence (AI) has emerged as a transformative tool in breast cancer diagnostics, leveraging machine learning (ML) and deep learning (DL) algorithms to enhance accuracy, efficiency, and accessibility. AI applications in digital mammography (DM), digital breast tomosynthesis (DBT), ultrasound, and magnetic resonance imaging (MRI) have demonstrated improved sensitivity and specificity, reducing false positives and false negatives while optimizing radiologist workload. Despite these advancements, challenges such as data accessibility, algorithm biases, regulatory constraints, and clinical integration hinder widespread AI adoption. Addressing these limitations requires standardized validation protocols, enhanced interpretability through explainable AI (XAI), and improved clinician and patient education. This editorial explores the evolving role of AI in breast cancer screening and diagnosis, emphasizing its potential to bridge healthcare disparities and improve global breast cancer outcomes.

## Editorial

Introduction

Breast cancer remains the most prevalent cancer globally, posing a significant public health challenge with both high incidence and mortality rates [1,2]. In 2020 alone, an estimated 2.3 million new cases were diagnosed, accounting for approximately 11.7% of all cancer diagnoses worldwide, with 685,000 deaths attributed to the disease [1-3]. One in every eight women will develop breast cancer during their lifetime, with the highest susceptibility observed among women aged 45-65 years due to hormonal changes associated with menopause [2]. The five-year survival rate for breast cancer varies significantly depending on the stage at diagnosis, ranging from 98% in stage I to only 24% in stage IV, highlighting the importance of timely detection and intervention [2].

Advancements in breast cancer screening and treatment have contributed to declining mortality rates in high-income countries, mainly due to the widespread adoption of mammography and improvements in therapeutic options [2]. Mammography remains the gold standard for breast cancer screening, with studies demonstrating its efficacy in reducing mortality by 9-15% [2]. The World Health Organization (WHO) has set ambitious targets to reduce breast cancer-related deaths by 25% by 2030 and to achieve an annual 2.5% decline in fatalities [2]. However, the effectiveness of screening programs is heavily dependent on participation rates and equitable access across diverse populations [2]. In low-resource settings, barriers such as limited access to screening, lack of specialized healthcare providers, and disparities in healthcare infrastructure contribute to late-stage diagnoses and poorer outcomes [4]. The WHO’s Global Breast Cancer Initiative (GBCI) was launched in 2021 to address these challenges, emphasizing early detection, prompt diagnosis, and treatment completion to improve survival rates [4].

Despite the effectiveness of mammography, traditional breast cancer screening methods remain constrained by human limitations [5]. Radiologists interpreting mammograms face challenges in distinguishing malignant lesions from benign abnormalities, often resulting in false positives that lead to unnecessary patient recall or false negatives that delay critical diagnoses [5]. Screening programs require substantial human resources, as radiologists must analyze vast numbers of images, most of which are normal [5]. In some countries, screening mammograms are reviewed by at least two radiologists to minimize errors, further increasing workload and operational costs [5]. Computer-aided detection (CAD) systems, first introduced in the 1990s using traditional machine learning (ML) techniques, were designed to assist radiologists by highlighting suspicious areas on mammograms [4,5]. However, their high false-positive rates led to limited adoption in many screening programs [5]. The advent of artificial intelligence (AI) has transformed medical imaging and diagnostics, offering new possibilities for improving breast cancer screening and treatment [5].

This editorial explores the transformative impact of AI in breast cancer screening and diagnosis. We will discuss how AI is revolutionizing detection accuracy and reducing diagnostic delays. Additionally, we will examine the potential barriers to AI adoption and strategies to overcome them. By leveraging AI technology effectively, the medical community can improve early detection, optimize treatment outcomes, and reduce global disparities in breast cancer care.

Artificial intelligence, machine learning, and deep learning

**Figure 1:**
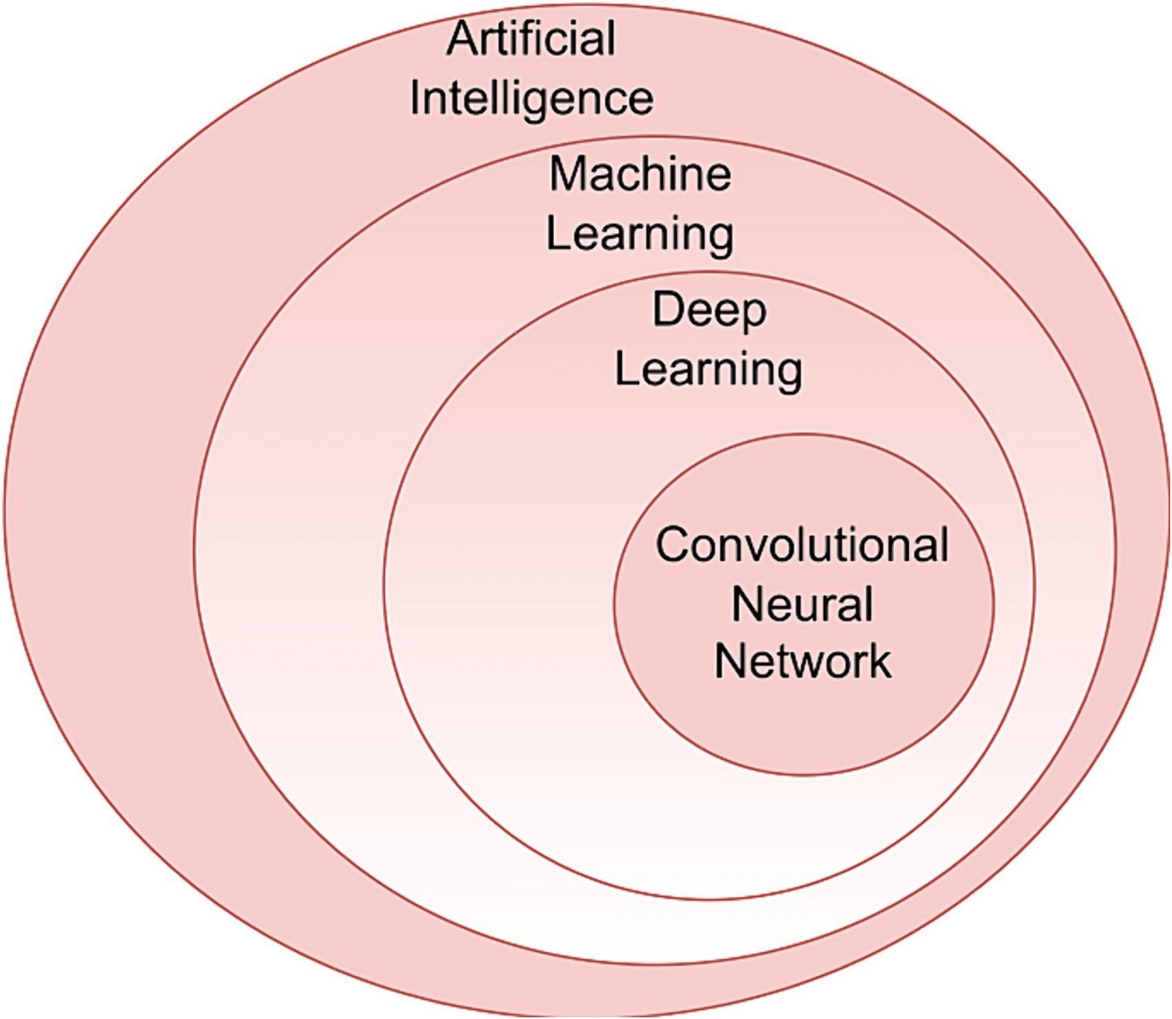
Diagram showing the artificial intelligence ecosystem with several of its subfields. Adapted with permission from Díaz et al. [5].

**Figure 2:**
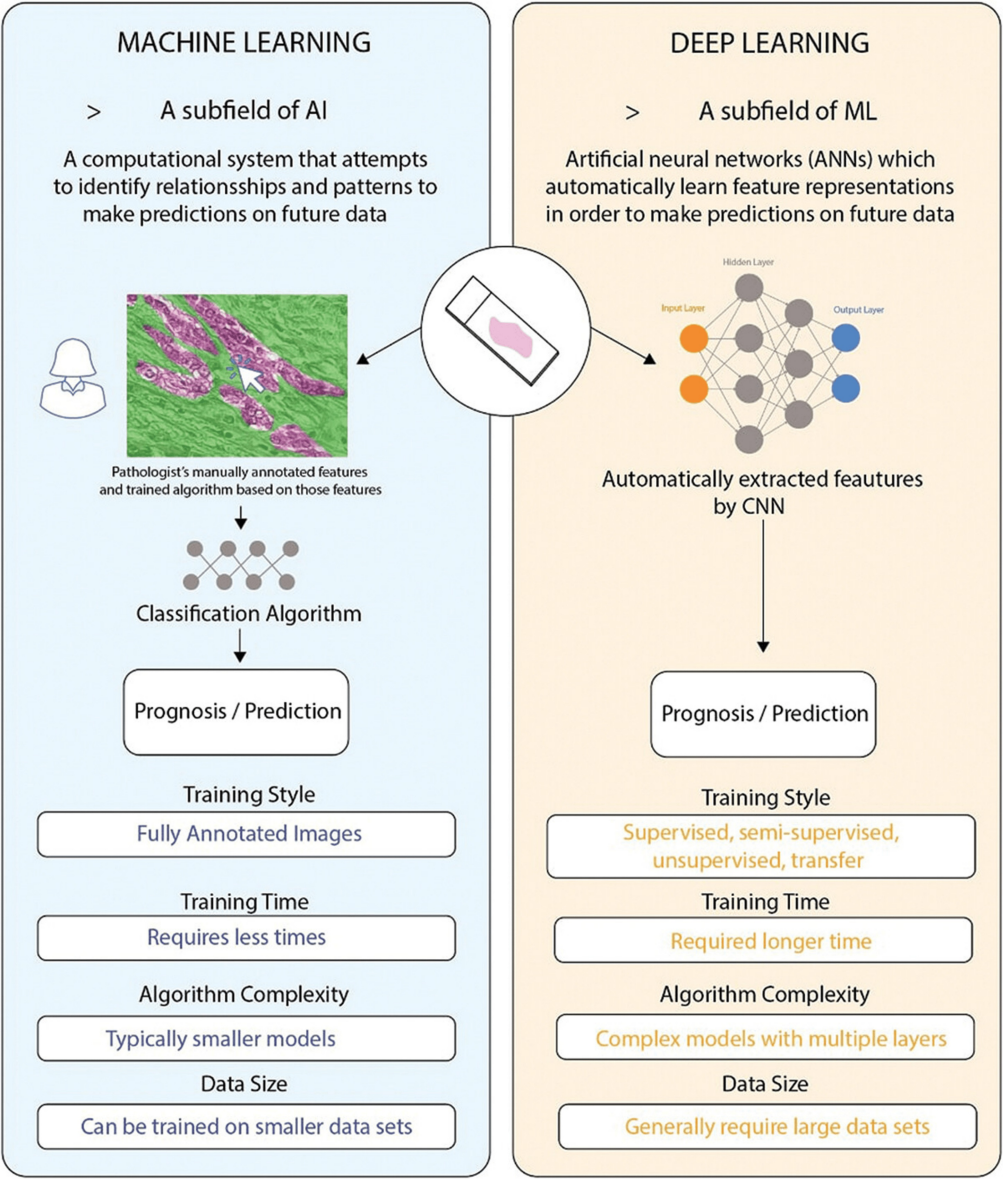
The differences between traditional machine learning (ML) and deep learning (DL). An input layer, an output layer, and multiple hidden layers make up convolutional networks. AI: artificial intelligence; CNN: convolutional neural network. Adapted with permission from McCaffrey et al. [3].

AI is a branch of computer science that aims to emulate human cognitive functions such as learning, problem-solving, and decision-making [3,5]. It encompasses various subfields, including ML and deep learning (DL) (Figure 1) [3,5]. ML involves computational systems that analyze vast datasets using algorithms to identify patterns and make predictions, improving performance over time through experience (Figure 2) [3]. DL, a more advanced subset of ML, relies on artificial neural networks (ANNs), which are designed to mimic the structure and function of the human brain [3,5]. These networks consist of multiple layers (input, hidden, and output layers), where each neuron processes information through weighted connections (Figure 2) [3]. Unlike traditional ML, DL does not require manual feature selection; instead, it automatically learns and refines feature representations through a process called backpropagation [3,5]. Convolutional neural networks (CNNs), a popular form of DL, are widely used in medical imaging, including breast cancer detection, due to their ability to analyze complex visual patterns with high accuracy [5]. As computational power continues to advance, AI, ML, and DL are increasingly applied in medical diagnostics, streamlining processes and enhancing decision-making accuracy [5].

AI in diagnosis and screening

AI has significantly advanced breast cancer screening and diagnosis by enhancing various imaging modalities [2]. In digital mammography (DM) and digital breast tomosynthesis (DBT), AI has improved radiologist’s accuracy and efficiency [5]. A study assessing an AI-based clinical decision support application for DBT showed that radiologists were able to increase sensitivity while maintaining specificity to decrease false negative interpretations without increasing benign biopsy recommendations [2]. Other studies have shown that AI-assisted interpretation increases the area under the curve (AUC) for radiologists, leading to more accurate cancer detection [2,5]. For example, AI integration with DM and DBT has demonstrated improvements in AUC from 0.769 to 0.797 and from 0.80 to 0.895, respectively, while also reducing workload in some cases [5]. AI is also being utilized as a stand-alone second reader, where it independently reviews screening mammograms. This approach has been shown to reduce recall rates while maintaining or improving cancer detection rates [5]. In one study, AI implementation resulted in a 4% increase in cancer detection while reducing recall rates by 4%, leading to a significant reduction in the number of screening readings required [5]. Similarly, AI used as a triage tool has enabled stratification of low- and high-risk cases, allowing for more efficient use of radiologists' time by prioritizing high-risk examinations for double reading [5].

AI has also significantly enhanced breast ultrasound imaging, improving lesion classification and reducing inter-reader variability [2]. AI-based decision support in breast ultrasound has demonstrated an increase in AUC from 0.83 to 0.87, allowing for more standardized and reliable assessments [2]. Ultrasound plays a vital role in breast cancer detection, particularly in identifying small, non-calcified lesions [2]. Emerging techniques, such as elastography and automated full-volume breast scan imaging, further enhance diagnostic accuracy [2]. DL models, including CNNs, have demonstrated up to 90% accuracy in distinguishing benign from malignant tumors [2]. Additionally, AI-assisted elastography has improved preoperative prediction of axillary lymph node metastasis, aiding in personalized treatment planning [2]. AI-assisted feature extraction and classification methods have further refined breast cancer detection, with some models reaching 99.1% accuracy [2]. However, ultrasound remains operator-dependent, requiring collaboration between ultrasonographers and AI systems for optimal performance [2]. Studies comparing AI algorithms to radiologists have yielded mixed results, with AI models reducing false positives and false negatives but not consistently outperforming human experts [2]. Combining AI with radiologists, however, has shown the most significant improvements in detection accuracy [2].

Additionally, AI is aiding in breast MRI analysis by automating lesion segmentation and classification, contributing to more precise and reproducible diagnoses [1]. Overall, AI is transforming breast cancer screening and diagnosis by improving accuracy, reducing workload, and optimizing workflow efficiency across multiple imaging modalities.

Challenges and limitations

Despite its potential, AI in breast cancer screening and diagnosis faces several challenges, primarily related to data availability, algorithm generalizability, regulatory concerns, and clinical adoption. Developing DL-based AI tools requires large, high-quality datasets that are diverse and representative of real-world populations [1,2]. However, access to such data remains limited due to privacy regulations, variations in imaging protocols, and disparities in healthcare infrastructure [1]. The inconsistency in datasets leads to biases in AI models, potentially affecting their reliability and applicability across different patient populations.

Another significant challenge is the lack of standardized image segmentation techniques [2]. AI-driven segmentation methods, such as thresholding, region-based, and clustering-based techniques, vary in accuracy and reproducibility [2]. Manual annotation by radiologists remains the gold standard, but it is labor-intensive, time-consuming, and prone to inter-observer variability [2]. Furthermore, the complexity of distinguishing small or ambiguous lesions from normal breast tissue can lead to inconsistent segmentation results, affecting the performance of AI models [2].

Furthermore, the integration of AI into clinical workflows raises ethical and regulatory concerns, particularly regarding patient privacy, data security, and liability [2]. AI systems require access to extensive medical data, increasing the risk of data breaches and unauthorized use [2]. Additionally, AI's "black-box" nature, where algorithms make decisions without clear explanations, can reduce clinician trust and hinder widespread adoption [1,3,5].

From a clinical perspective, AI adoption depends on how seamlessly it integrates into existing diagnostic pathways. In ultrasound imaging, for instance, AI performance heavily depends on operator input, creating variability in diagnostic accuracy [2]. Unlike CT and MRI, where image acquisition is more standardized, ultrasound imaging requires real-time interpretation, necessitating close collaboration between ultrasonographers and AI systems [2]. This dependence on human input slows AI adoption in ultrasound compared to other imaging modalities.

Mammography, a cornerstone of breast cancer screening, has benefited from AI, yet concerns regarding overdiagnosis persist [2]. While AI can enhance early detection, it may also increase the identification of indolent lesions, leading to unnecessary interventions and financial burdens.

Future directions

Advancing AI Standalone Performance

Recent advancements suggest that AI can potentially function as a stand-alone reader for breast cancer screening, reducing the burden on radiologists [2]. However, randomized controlled trials (RCTs) directly comparing AI to human interpretation are still lacking [2]. Prospective studies validating AI performance in real-world settings are essential before widespread clinical adoption.

Policy Changes

Regulatory agencies must refine guidelines to ensure AI tools used in breast cancer screening and treatment meet high standards of safety and efficacy. The U.S. Food and Drug Administration (FDA) and European regulatory bodies should establish robust frameworks that emphasize transparent validation methods, real-world performance assessments, and post-marketing surveillance to ensure ongoing safety and effectiveness. Improved regulatory oversight will be critical in maximizing the benefits of AI adoption while minimizing unintended risks.

To enhance trust in AI-driven imaging, well-established guidelines and checklists widely accepted by the medical imaging community should be implemented. Traceability measures must be incorporated alongside mechanisms for documenting and monitoring the development and real-world functioning of AI tools in clinical settings. Increased transparency, such as the disclosure of datasets used to train and validate AI algorithms, will improve quality assurance and assist clinicians in making informed decisions when adopting these technologies. Additionally, internal validation using local data is essential to detect potential errors or biases, such as those arising from training with imaging systems from a single vendor. These policy refinements will be crucial in ensuring AI-driven innovations align with clinical needs while maintaining safety, reliability, and fairness.

Enhancing AI Interpretability and Usability for Clinicians

To gain clinician trust, AI must become more interpretable. Current DL models often lack transparency, making it difficult for radiologists to understand how AI reaches its conclusions. Explainable AI (XAI) techniques, such as saliency maps and gradient-weighted class activation mapping (Grad-CAM), can help bridge this gap by visualizing AI decision-making processes [1]. Future research should focus on integrating XAI into clinical workflows to provide radiologists with interpretable and actionable insights.

Additionally, AI interfaces should be designed for seamless integration with radiology workstations and electronic health records (EHRs), ensuring that AI-generated recommendations are easily accessible and clinically relevant. AI tools should not only detect cancer but also provide risk assessments, suggest follow-up protocols, and integrate with multidisciplinary decision-making processes.

Educating Patients and Clinicians

Public perception of AI in healthcare is evolving, with many patients expressing confidence in AI-assisted decision-making [2]. However, AI adoption in breast cancer care requires public education to ensure informed consent and patient engagement. Transparent communication about AI’s benefits, limitations, and role in shared decision-making is crucial to building trust. Clinician education is equally important. Medical schools and residency programs should incorporate AI literacy into their curricula, equipping future radiologists and oncologists with the skills needed to interpret AI-generated insights. Continuing medical education (CME) programs should also provide training on AI integration in clinical practice.

Conclusion

The integration of AI into breast cancer screening and treatment represents a significant advancement in oncologic care. AI-driven technologies have demonstrated the ability to enhance diagnostic accuracy, reduce radiologist workload, and optimize early detection, ultimately improving patient outcomes. From ML algorithms aiding in mammography interpretation to DL models refining ultrasound and MRI assessments, AI is reshaping the landscape of breast cancer diagnostics. However, despite these promising advancements, challenges such as data availability, algorithm biases, regulatory hurdles, and clinical adoption barriers remain. Ensuring equitable access to AI-powered screening, fostering clinician trust through XAI, and refining regulatory frameworks will be essential in maximizing AI’s potential. Moving forward, a collaborative effort between researchers, healthcare professionals, and policymakers is necessary to ensure AI is implemented responsibly, improving the efficiency and accessibility of breast cancer care worldwide. By leveraging AI technology effectively, the medical community can bridge existing disparities, enhance early detection, and ultimately reduce breast cancer mortality on a global scale.
